# New records of *Paraleptophlebia werneri* Ulmer, 1920 and *Paraleptophlebia strandii* (Eaton, 1901) from Finland (Ephemeroptera, Leptophlebiidae)

**DOI:** 10.3897/BDJ.1.e969

**Published:** 2013-09-16

**Authors:** Jukka Salmela, Eino Savolainen

**Affiliations:** † Natural Heritage Services (Metsähallitus), Rovanieni, Finland; ‡ University of Turku, Zoological Museum, Turku, Finland; § Natural History Museum of Kuopio, Kuopio, Finland

**Keywords:** Lapland, faunistics, mayflies, aapamires, ponds

## Abstract

The mayfly species *Paraleptophlebia werneri* has been rediscovered from Finland. The species was classified as RE (regionally extinct) in the most recent national red-list assessment. The new locality is close to the Russian border in NE Lapland, Savukoski. Adult males were collected with a sweep net around a pond. *Paraleptophlebia strandii* is a rather poorly known but widespread Finnish species. The adults of this species occurred in great numbers in aapamires of central Lapland (Sodankylä). We hypothesize that these leptophlebid species are not dependent on running water but may instead thrive in small lentic water bodies.

## Introduction

The genus *Paraleptophlebia* Lestage (Ephemeroptera, Leptophlebiidae) consists of six European species (Bauernfeind and Soldán 2012), four of which have been recorded from Finland (*Paraleptophlebia cincta* (Retzius, 1783), *Paraleptophlebia strandii* (Eaton, 1901), *Paraleptophlebia submarginata* (Stephens, 1835) and *Paraleptophlebia werneri* Ulmer, 1920, Savolainen 2009). According to Savolainen (2009), larvae of the Finnish species are associated with lotic waters. Some species, however, have been collected from summer-dry streams and slow-flowing parts of rivers (Bauernfeind and Soldán 2012). In this paper we report two poorly known and rarely caught species based on material collected from Finnish Lapland, north boreal ecoregion. Brief historical and ecological accounts are given for the species.

## Materials and methods

Adult specimens of mayflies were collected by using a sweep net in 2012 (Suppl. materials 1, 2). Specimens were collected among aerial swarms or from lower vegetation. The collected sample was placed in a plastic bag with a pad wetted in chloroform and stunned insects were finally preserved in ethanol. Mayfly nymphs were searched by using kick-net (mesh size 0,5 mm) in 2013. Layer photos were taken using an Olympus E520 digital camera attached to an Olympus SZX16 stereomicroscope. Digital photos were captured using the softwareDeep Focus v. 3.1 and Quick PHOTO CAMERA 2.3. Layer photos were finally combined with the program Combine ZP. Coordinates are given in WGS84 format.

## Taxon treatments

### Paraleptophlebia werneri

Ulmer, 1920

#### Materials

- **Type status:** Other material. **Occurrence:** recordedBy: J. Salmela; individualCount: 2; sex: males; lifeStage: adult; **Location:** country: Finland; verbatimLocality: Lapponia kemensis pars orientalis: Savukoski, Törmäoja, Ahot; verbatimLatitude: 67.8276 N; verbatimLongitude: 29.4394 E; **Event:** eventDate: 16.8.2012; **Record Level:** institutionCode: Jukka Salmela
- **Type status:** Other material. **Occurrence:** catalogNumber: 8607; recordedBy: J. Salmela; individualCount: 7; sex: males; lifeStage: adult; **Location:** country: Finland; verbatimLocality: Lapponia kemensis pars orientalis: Savukoski, Törmäoja, Ahot; verbatimLatitude: 67.8276 N; verbatimLongitude: 29.4394 E; **Event:** eventDate: 16.8.2012; **Record Level:** institutionCode: Kuopio Natural History Museum

#### Notes

**Figure 1.**
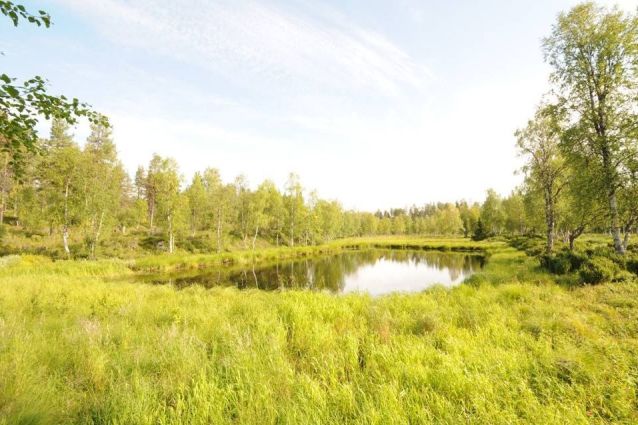
The collecting locality of *Paraleptophlebia werneri* in NE Lapland, Savukoski, Törmäoja in 16.8.2012. Photo J. Ilmonen.

The sampling locality was a pond in a rather open landscape (Fig. 1). The pond is apparently permanent, i.e. not very susceptible of drying out in summer. Its surface area was ca. 850 m^2^ and its maximum depth ca. 150 cm. The water was slightly brownish, colored by humic substances. There was sparse submerged vegetation, shores were wet and dominated by tall sedges and grasses. The pond was likely lacking fish and without an outlet, except for a marshy area connecting it to Törmäoja stream. The distance from Törmäoja stream was ca. 80 m. The adult males were caught from the shores of the pond by using a sweep net. No other mayfly species were present in the sample. In 2012 a sweep net sample was also collected from the shore of the stream, some 900 m north of the pond, but no *Paraleptophlebia werneri* specimens were found. The sampling locality was a pond in a rather open landscape (Fig. 1). The pond is apparently permanent, i.e. not very susceptible of drying out in summer. Its surface area was ca. 850 m^2^ and its maximum depth ca. 150 cm. The water was slightly brownish, colored by humic substances. There was sparse submerged vegetation, shores were wet and dominated by tall sedges and grasses. The pond was likely lacking fish and without an outlet, except for a marshy area connecting it to Törmäoja stream. The distance from Törmäoja stream was ca. 80 m. The adult males were caught from the shores of the pond by using a sweep net. No other mayfly species were present in the sample. In 2012, a sweep net sample was also collected from the shore of the stream, some 900 m north of the pond, but no P. werneri specimens were found. This streamside sample included two mayfly species: *Paraleptophlebia cincta* (Retzius, 1783) and *Siphlonurus lacustris* Eaton, 1870. However, in 2013 one *Paraleptophlebia werneri* male specimen was caught from the slow flowing section of the headwater stream, some 470 m north of the pond. Larvae of *Paraleptophlebia werneri* were collected from the bottom of the pond, among fine organic detritus and submerged vegetation. Larvae of this species were also collected from a nearby permanent pond, surface area 830 m^2^, lacking inlet or outlet brooks. Geographic distance of these two ponds is 760 meters. Two other smaller, temporary ponds in the vicinity were also sampled but no ephemeropteran larvae were found.

### Paraleptophlebia strandii

(Eaton, 1901)

#### Materials

- **Type status:** Other material. **Occurrence:** recordedBy: J. Salmela; individualCount: 5; sex: males; **Location:** verbatimLocality: Lapponia kemensis pars orientalis: Sodankylä, Satovaara; locationRemarks: rich flark fen; verbatimLatitude: 67.6777 N; verbatimLongitude: 27.0878 E; **Event:** eventDate: 20.8.2012
- **Type status:** Other material. **Occurrence:** recordedBy: J. Salmela; individualCount: 5; sex: males; **Location:** verbatimLocality: Lapponia kemensis pars orientalis: Sodankylä, Sonniharju SE; locationRemarks: rich flark fen; verbatimLatitude: 67.6823 N; verbatimLongitude: 27.0926 E; **Event:** eventDate: 20.8.2012
- **Type status:** Other material. **Occurrence:** recordedBy: J. Salmela; individualCount: 10; sex: males; **Location:** verbatimLocality: Lapponia kemensis pars orientalis: Sodankylä, Postoselkä E; locationRemarks: rich flark fen; verbatimLatitude: 67.7978 N; verbatimLongitude: 26.6655 E; **Event:** eventDate: 9.8.2012

#### Notes

All sampling localities are aapamires, i.e. fens that receive water and nutrient input from the surrounding mineral lands (versus raised bogs that only receive rain water). The mires are lying on bedrock which consists of calcareous or non-acidic rocks such as ultramafic volcanic rock, gabbro and schists (Geological Survey of Finland, http://en.gtk.fi/). The calcareous influence can be seen in the bryophyte flora, which includes species such as *Campylium stellatum*, *Scorpidium revolvens*, *Tomentypnum nitens* and *Hamatocaulis vernicosus*. The fens were characterized by wet flarks (i.e. inundated moss cover) and mud-bottom pools. The collecting sites were hard to access and difficult to walk on. There were no available mineral substrates for aquatic insects. However, due to their wetness and sloping profile (most notably in Sonniharju), there were some shallow surface flows on the fens.

Adults of *Paraleptophlebia strandii* were very numerous at the collecting sites. Males formed huge swarms consisting of thousands of specimens. Swarming took place over narrow strings with sedge (*Carex*) shoots in the vicinity of the pools. Swarming was observed approximately within 0.6 and 2 m of height, in the afternoon during sunny weather. The minimum distance to the nearest stream was one kilometer.

## Discussion

*Paraleptophlebia werneri* is awestern Palaearctic species. Its range extends from the British Isles to the eastern side of Urals (Russia), and from northern Fennoscandia to the Alps (Bauernfeind and Soldán 2012). The species occurs in Norway (Johansen and Lunde 1993) and the Swedish records are from the northern provinces (Lycksele Lappmark, Pite Lappmark and Torne Lappmark, Engblom 2001). In his nationwide mayfly survey, Tiensuu (1939) reported only a single locality for *Paraleptophlebia werneri* from *Regio kuusamoensis*: Kuusamo, W. Hellén leg., 1 male. No more specific locality or habitat information was given in that publication or found in Tiensuu’s otherwise detailed note books. It is likely however that the locality is currently on the Russian side of the border (Savolainen 2009). Another verified record is the one by Mauri Hirvenoja from *Lapponia kemensis pars orientalis*: Sodankylä, Mutenianjoki River, 30.6.1960, several swarming males (Savolainen 2009). One of us (ES) saw the sample and verified the identification in 1967. Regrettably, the Mutenianjoki river and the surrounding landscape were inundated and destroyed at the end of the 1960s due to the construction of the Lokka reservoir (200-400 square kilometers in area, depending on the water level). In addition to the above mentioned two sites, the species was reported from South Finland (*Tavastia australis*: Hausjärvi, [Hirvenoja 2002]). However, that record was based on small larvae and the habitat has since been destroyed (M. Hirvenoja, personal communication). In order to assess its range size and conservation status, *Paraleptophlebia werneri* has been the focus of intensive searches during the last four decades in North Finland. Nevertheless, the species remained undetected until 2012. In the most recent national red-list assessment *Paraleptophlebia werneri* was classified as RE (regionally extinct, Savolainen and Ilmonen 2010).

Adult males of *Paraleptophlebia werneri* are easily distinguished from other European species due to their characteristic genitalia and the shape of their gonopods (Fig. 2). See, e.g., Elliott and Humpesch (1983) and Bauernfeind and Soldán (2012) for the identification of adults; Macan (1979) and Bauernfeind and Humpesch (2001) for the identification of larvae.

According to literature records the larvae of this species dwell in several kinds of water bodies. Bauernfeind and Soldán (2012) state that *Paraleptophlebia werneri* is an inhabitant of lowland streams and rivers characterized by abundance of submerged vegetation, usually in backwaters and ephemeral (summer dry) water bodies. Also Elliott and Humpesch (1983) note that the species thrives in lotic waters that lack water flow during summer. In the Swedish fell area the species has been caught from alpine headwater streams and even small ponds (Engblom 2001, E. Engblom, personal communication).

The first Finnish locality, the now vanished Mutenianjoki river, was a less than 10 m in breadth, eutrophic, slow flowing river with a muddy and stony bed. During summer time the river usually had low water level. The water was neutral or alkaline (pH over 7) and turbid due to abundance of algae. The river harbored aquatic insect larvae in great numbers. Mauri Hirvenoja collected his sample from the slow flowing outlet of the river. Lake Sompiojärvi, the source of the river, was shallow but well oxygenated (M. Hirvenoja, personal communication).

According to Bauernfeind and Soldán (2012) *Paraleptophlebia werneri* is a univoltine, early summer species, whose adults emerge from April to June. The record from River Mutenianjoki is from the end of June. In northern Sweden, however, larvae have been collected at the end of July (E. Engblom, personal communication). The adult males reported here were caught in mid August.

*Paraleptophlebia strandii* is a North Palaearctic species with a wide range encompassing Fennoscandia and Russian Far East (Bauernfeind and Soldán 2012). The first Finnish record was given by Aro (1928), from three provinces in southern and northern Finland. According to Savolainen (2009) the species is absent from western and southwestern Finland and has been recorded from eastern and northern Finland, including northernmost Lapland.

The natural historyand occurrence of *Paraleptophlebia strandii* are hitherto poorly known. There are scanty notes in the literature (see Bauernfeind and Soldán 2012), and according to Tiensuu (1935) the species favors small forest brooks with a slow current. In Finland the species is associated with running waters, especially small streams with aquatic mosses and vascular plants (Savolainen 2009). The life cycle of the species is unknown but it may be of the univoltine summer type (Bauernfeind and Soldán 2012).

In the present work were report *Paraleptophlebia strandii* from boreal aapamires. These new observations are significant, because the notion of the species as a lotic one is challenged. Based on earlier records this species occurs in running water habitats with high oxygen levels. Our new records indicate that *Paraleptophlebia strandii* is a common inhabitant of northern aapamires and that the species may be locally abundant. It may be assumed that *Paraleptophlebia strandii* overwinters as an egg. Due to flowing of groundwater and thick snow cover, the flarks and pools in aapamires may retain some free water during snow covered periods (Koutaniemi and Seppälä 1986, Seppälä and Koutaniemi 1985), thus enabling the survival of the eggs. On the other hand, it has been noted that eggs of other mayfly species such as *Baetis macani* and *Baetis bundyae* may tolerate freezing (Giberson et al. 2007, Drotz et al. 2012).

## Supplementary Material

XML Treatment for Paraleptophlebia werneri

XML Treatment for Paraleptophlebia strandii

## Figures and Tables

**Figure 2a.**
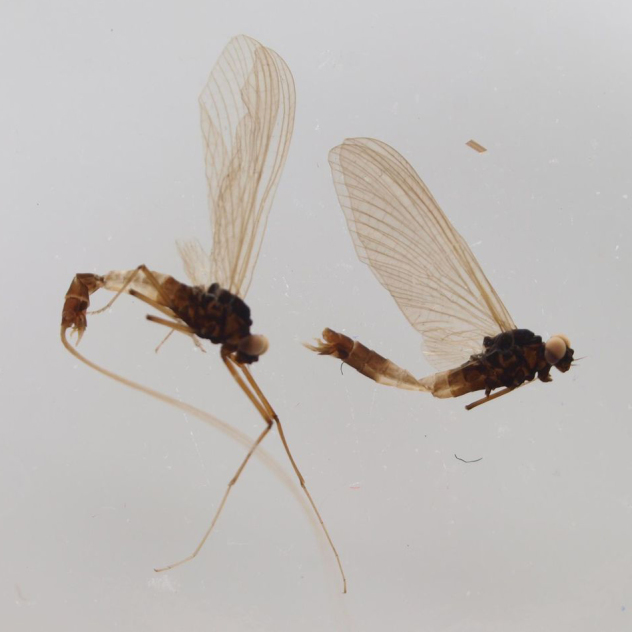
adult males, habitus, lateral view, wing length ca. 5.5 mm

**Figure 2b.**
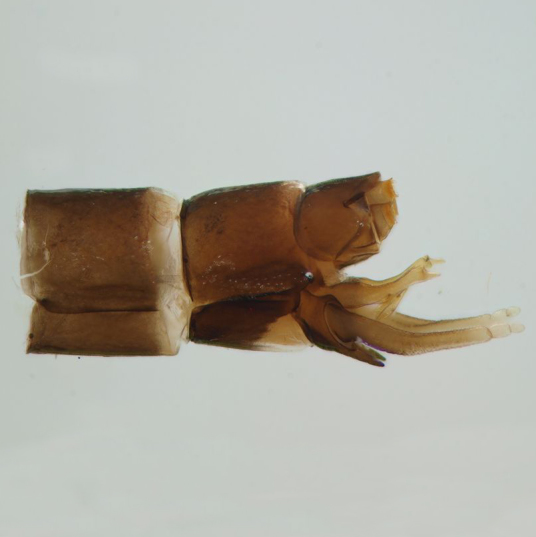
male abdominal terminalia, lateral view

**Figure 2c.**
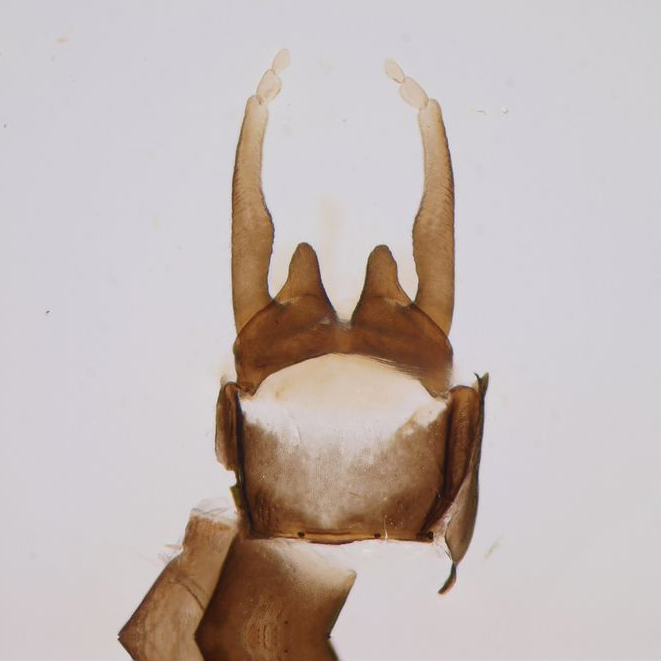
male abdominal terminalia and forceps (gonopods), ventral view

**Figure 2d.**
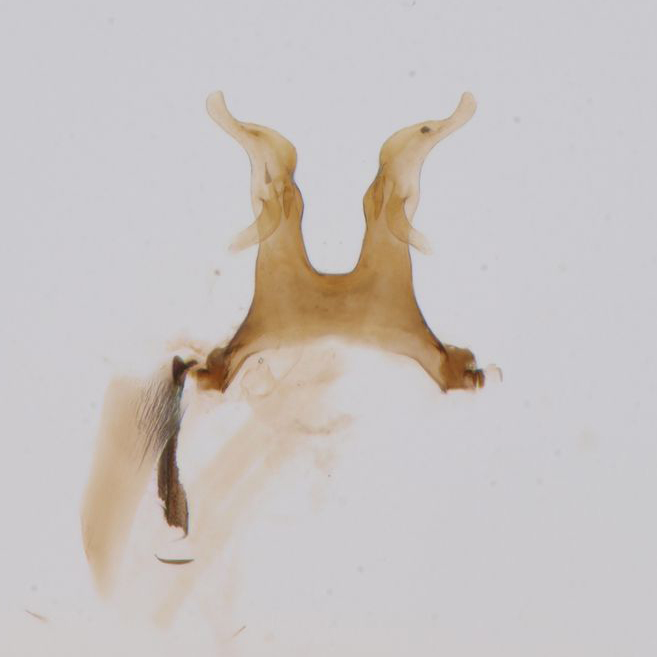
penis, ventral view

## Supplementary files

### New records of Paraleptophlebia werneri and Paraleptophlebia strandii from Finland

**Authors:** J. Salmela & E. Savolainen **Data type:** occurences New records of four may fly species are given from Finnish Lapland **File:** occurrence-1_Paraleptophlebia.xlsx

### Occurrence data

**Authors:** J. Salmela & E. Savolainen **Data type:** occurences **File:** occurrence-1_Paraleptophlebia.xlsx
